# Computational Simulation Model to Predict Behavior Changes in Inflammatory Bowel Disease Patients during the COVID-19 Pandemic: Analysis of Two Regional Japanese Populations

**DOI:** 10.3390/jcm12030757

**Published:** 2023-01-18

**Authors:** Gen Suzuki, Ryuichi Iwakiri, Eri Udagawa, Sindy Ma, Ryoko Takayama, Hiroshi Nishiura, Koshi Nakamura, Samuel P. Burns, Paul Michael D’Alessandro, Jovelle Fernandez

**Affiliations:** 1 Japan Medical Office, Takeda Pharmaceutical Company Limited, Tokyo 103-8668, Japan; 2 PricewaterhouseCoopers Advisory Services LLC, Philadelphia, PA 19103, USA; 3 PricewaterhouseCoopers Consulting LLC, Tokyo 100-0004, Japan; 4 Health & Environment Science, Kyoto University, Kyoto 606-8601, Japan; 5 Department of Public Health and Hygiene, Graduate School of Medicine, University of the Ryukyus, Okinawa 903-0215, Japan

**Keywords:** COVID-19, pandemic, inflammatory bowel disease, ulcerative colitis, Crohn’s disease, biologics, computer simulation

## Abstract

Managing inflammatory bowel disease (IBD) is a major challenge for physicians and patients during the COVID-19 pandemic. To understand the impact of the pandemic on patient behaviors and disruptions in medical care, we used a combination of population-based modeling, system dynamics simulation, and linear optimization. Synthetic IBD populations in Tokyo and Hokkaido were created by localizing an existing US-based synthetic IBD population using data from the Ministry of Health, Labor, and Welfare in Japan. A clinical pathway of IBD-specific disease progression was constructed and calibrated using longitudinal claims data from JMDC Inc for patients with IBD before and during the COVID-19 pandemic. Key points considered for disruptions in patient behavior (demand) and medical care (supply) were diagnosis of new patients, clinic visits for new patients seeking care and diagnosed patients receiving continuous care, number of procedures, and the interval between procedures or biologic prescriptions. COVID-19 had a large initial impact and subsequent smaller impacts on demand and supply despite higher infection rates. Our population model (Behavior Predictor) and patient treatment simulation model (Demand Simulator) represent the dynamics of clinical care demand among patients with IBD in Japan, both in recapitulating historical demand curves and simulating future demand during disruption scenarios, such as pandemic, earthquake, and economic crisis.

## 1. Introduction

The COVID-19 outbreak caused by severe acute respiratory syndrome coronavirus 2 (SARS-CoV-2) was first reported in December 2019 and emerged as a global pandemic. Data from the COVID-19 dashboard on 30 September 2021 reported more than 232 million cases of COVID-19 infection across 220 countries with over 4.7 million deaths [1]. The high rate of transmission, frequent need for hospitalization, and demand for intensive-care management has led to a rapid change in the organization of healthcare systems around the world [2,3]. Government-mandated lockdowns and stay-at-home orders have resulted in reduced utilization of healthcare services [4].

Inflammatory bowel diseases (IBD), such as ulcerative colitis (UC) and Crohn’s disease (CD), are chronic inflammatory conditions of the gastrointestinal tract (GI) affecting millions of people worldwide [5]. Standard-of-care includes regular follow-up with bio-marker monitoring (e.g., fecal calprotectin, C-reactive protein), endoscopy, and cross-sectional imaging [6]. More than 50% of patients with CD develop complications that require surgery, and 20–30% of patients with UC undergo colectomy [6]. Commonly, IBD is treated with immunosuppressive therapies including corticosteroids, immunomodulators, biologic agents including monoclonal antibody inhibitors of tumor necrosis factor (TNF) alpha, interleukin 12/23, integrins, and small molecules such as Janus kinase (JAK) inhibitors [7].

The restructuring of healthcare systems to treat patients with COVID-19 and prevent transmission of the virus has drastically reduced non-essential clinical activities for IBD care [3,8]. The full impact has yet to be realized from issues such as reduced access to diagnostic endoscopy, lack of face-to-face clinics, difficulties in continuing day-case infusions, and disruption in performing routine blood and/or stool monitoring, in addition to patients’ behaviors, often manifesting themselves in fears that may have reduced their attendance in hospitals [3].

Experts on IBD have provided guidance on the management of IBD during the COVID-19 pandemic with respect to risk of infection, treatment with immunosuppressive therapies, use of medical services, and COVID-19 vaccination [7,9,10,11,12,13,14]. Evidence from a systematic review and meta-analysis of eight studies in over 9000 patients with IBD has indicated that the incidence of COVID-19 in the IBD population is no different to that of the general population (0.3% and 0.2–4.0%, respectively), implying that patients with IBD are not at a greater risk of COVID-19 infection [15]. A large international registry study (Surveillance Epidemiology of Coronavirus Under Research Exclusion for Inflammatory Bowel Disease: SECURE-IBD) has found that older age, increased number of comorbidities, and systemic corticosteroid use are strong risk factors for adverse outcomes of COVID-19 infection among patients with IBD [16,17]. Notably, TNF inhibitors were not associated with severe COVID-19 infection [16]. In addition, further analyses of data from the SECURE-IBD registry indicated that monotherapy with TNF inhibitors may be associated with a lower risk of COVID-19-associated hospitalization or death than other immunomodulatory treatment regimens (methotrexate or azathioprine/6-marcaptopurine with or without TNF inhibitors), with the exception of monotherapy with Janus kinase inhibitors [18].

Although the incidence of COVID-19 in the IBD population was found to be no greater than that of the general population, the disruption to healthcare services has caused significant reductions in capacity, deferred schedules, and delayed access to clinic visits, colonoscopies, and non-urgent surgeries [3,12]. Adaptive approaches made available during the pandemic to maintain appropriate IBD care have included remote monitoring, home delivery of medications, and patient education initiatives [12].

Healthcare disruption caused by COVID-19 has created a unique opportunity to gain insights into patient behavior and demand for healthcare services during an adverse situation. Understanding the impact of disruptions associated with the pandemic on patient behavior and healthcare supply will enable healthcare providers and administrators to better prepare for future crises. Therefore, we hypothesize that mathematical modeling using synthetic patient populations, systems dynamics modeling, computer simulation, and models to predict patients’ reactions in different disruptive scenarios can be used to examine such behavioral changes.

The purpose of this study was to understand the impact of the COVID-19 pandemic on patient behaviors and disruptions in medical care using a proof-of-concept (PoC) model and populations of patients with IBD in two regions of Japan.

## 2. Materials and Methods

**Figure 1:**
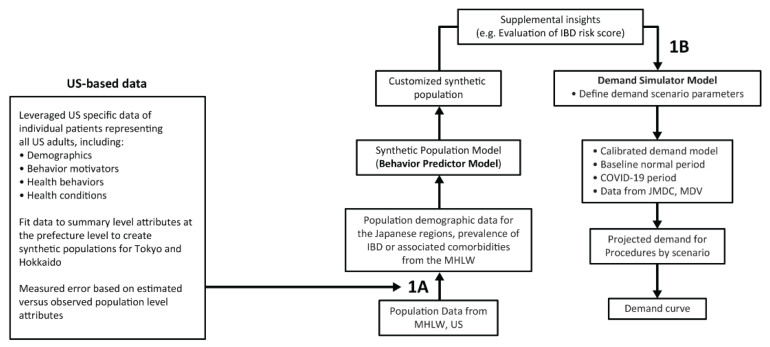
Creation of a Japanese-specific synthetic Behavior Predictor model (1A) and a Demand Simulator model (1B). IBD = inflammatory bowel disease; MDV = Medical Data Vision; MHLW = Ministry of Health, Labor and Welfare.

Analyses were based on a virtual synthetic IBD population model (Behavior Predictor) and a healthcare demand simulation model (Demand Simulator) (Figure 1).

### 2.1. Synthetic IBD Population Model

The Behavior Predictor model managed the synthetic population localization process for input into the demand simulation. It leveraged a US-based synthetic consumer dataset (derived from proprietary information of PwC) to represent Japanese patients with IBD configured using specific demographic, behavioral, and health condition prevalence data derived from the Japanese Ministry of Health, Labor and Welfare (MHLW) Health Statistics Office for 2016 (https://www.mhlw.go.jp/toukei/itiran/gaiyo/k-eisei.html, accessed on 1 October 2020, and https://www.e-stat.go.jp, accessed 1 October 2020). Patients with IBD aged 18 to 74 years in Tokyo and Hokkaido were selected to represent patients with IBD in a high-density urban mainland setting and regional island setting of Japan, respectively. The determination of an IBD diagnosis came from the claims data, and it was not known if genetic screening was utilized. If genetic information about the IBD population were known, it could be used to develop better patient-level models of disease progression for integration into the simulation model [19].

The US-based synthetic population (derived from proprietary information of PwC) included data on care utilization, health conditions, behaviors, information and content preferences, product choices and usage, and motivators and stressors. These data were collected and created from a combination of consumer, geospatial, and behavioral surveillance data via statistical matching algorithms. A linear programming approach was applied to optimize for a minimal difference between the synthetic and MHLW population. Outputs from this approach confirmed a low error between aggregate synthetic population model distributions versus Japanese-specific aggregate data on demographics, selected behaviors (such as smoking and alcohol consumption), and health states (see Supplemental Model Methodology for additional description [20,21]).

Data specific to COVID-19 infection, including the number of new positive cases, disease severity, and cumulative deaths, were derived from online data available from the Tokyo COVID-19 information site (https://stopcovid19.metro.tokyo.lg.jp/en, accessed on 1 October 2020) and the Hokkaido government site (http://www.pref.hokkaido.lg.jp/ss/dtf/opendata/covid19.htm, accessed on 1 October 2020). Data were collected for the pre-COVID-19 period from January 2018 to 10 March 2020 and for the post-COVID-19 period from 11 March 2020 (the day when the World Health Organization declared COVID-19 a global pandemic) to October 2020.

### 2.2. Demand Simulation Model

A clinical pathway of IBD-specific disease progression and simulation of the patient treatment experience through time was constructed in the Demand Simulator (agent-based system dynamics) model. The model included clinical pathways for asymptomatic patients not on treatment, symptomatic patients seeking care, and patients with mild-to-moderate or moderate-to-severe IBD who received continuous care (Supplementary Figure S1).

The Demand Simulator model was calibrated using longitudinal claims data for patients with IBD in Tokyo and Hokkaido obtained from Medical Data Vision (MDV) and JMDC Inc. Two sets of simulation model parameters were generated using data from January 2015 to December 2019 for IBD-related healthcare demand prior to the onset of COVID-19 and using data from January 2020 to October 2020 for demand during the COVID-19 pandemic. Future patient behavioral changes were simulated for up to 3 years (i.e., from November 2020 to October 2023) to assess the impact of disruption related to the COVID-19 pandemic (see Supplemental Model Methodology for additional description). This simulation model does not predict future COVID-19 pandemic prevalence but takes as input different scenarios that qualitatively reflect likely future trajectories of the pandemic in Japan. For this reason, the projections described are intended to show the reaction of the healthcare system to the proposed idealized scenarios. These projections are not expected to forecast the actual trajectory of COVID-19 prevalence in Japan and may not resemble data about the pandemic that become available after this study. Lists of input and output data for the demand simulation model are summarized in Supplementary Tables S1 and S2.

### 2.3. Simulation of Possible Future Crisis Scenarios for COVID-19 on Healthcare Demand

**Figure 2:**
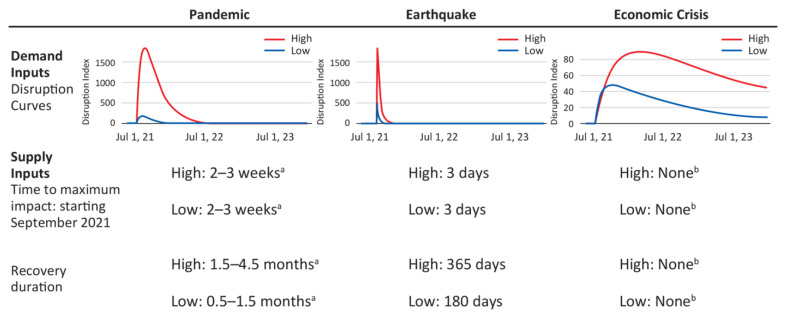
Disruption scenarios for pandemic, earthquake, and economic crisis. ^a^ Recovery durations based on historically observed values from the COVID-19 pandemic were used. ^b^ Economic crisis scenario is assumed to be purely a function of demand side disruptions.

The calibrated Behavior Predictor and Demand Simulator models were used to explore the impact of three crisis scenarios on IBD clinical treatment pathways: future periods of high COVID-19 incidence in Japan, an earthquake, and a financial crisis (Figure 2). Historical data were used to determine the scenario parameters for disruption due to COVID-19, but not for the earthquake and financial crisis scenarios. Input parameters for earthquakes and financial crises were determined based on the following assumptions: (1) logic (e.g., initial impact from earthquake is immediate) and (2) estimated impact relative to the COVID-19 response from consumers. A total of 1800 simulations were run to capture random effects and understand the impact of crisis disruptions on patients with IBD in Japan. Output metrics included patient counts by geography, sex, age, disease state, treatment stage, disease severity, and UC or CD; and disruption impact on supply and demand including diagnostic procedures and monitoring, number of vedolizumab infusions, and new patients starting vedolizumab.

### 2.4. Interventions to Address the Impact of Disruptions

The impact of recovery interventions designed to address disruptions to the IBD treatment pathway were assessed using the Behavior Prediction and Demand Simulator models to identify leverage points that can drive a return to normalcy (Table 1). All key interventions were related to healthcare services including communication with patients and transportation of medical supplies. Input metrics for each intervention were determined based on logic, estimated impact relative to COVID-19 disruption, and complexity of implementation. Interventions with values of 5–15% require individual involvement and have small-scale reach, whereas interventions with values of 55–60% require high-level involvement and large-scale delivery.

## 3. Results

### 3.1. Population Statistics

**Figure 3:**
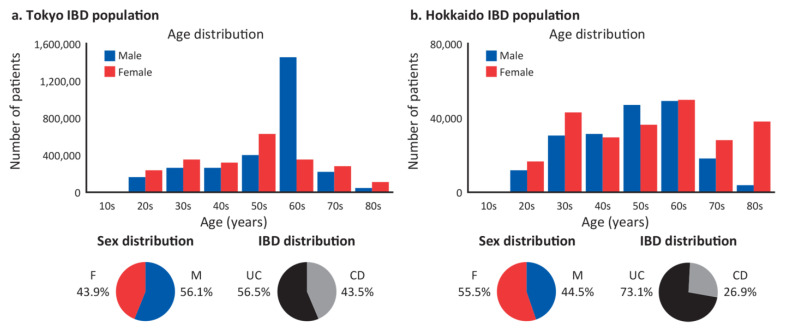
Demographic characteristics of synthetic patient populations for (**a**) Tokyo and (**b**) Hokkaido. CD = Crohn’s disease; IBD = inflammatory bowel disease; UC = ulcerative colitis.

The synthetic Japanese IBD population generated by the Behavior Predictor model was informed by demographic characteristics from the MHLW database and complemented with behavioral insights from the JMDC database. The resulting population included 4,873,585 patients with IBD (male/female, 56.1%/43.9%) for Tokyo and 438,983 patients with IBD (male/female, 44.5%/55.5%) for Hokkaido (Figure 3). The prevalence of UC was slightly higher than CD in the Tokyo population (56.5% vs. 43.5%) and substantially higher in the Hokkaido population (73.1% vs. 26.9%). Male and female patients with UC in the Tokyo population had a similar mean (standard deviation [SD]) age of 52.7 (15.5) years and 52.0 (15.6) years, respectively. However, female patients were slightly older than male patients in the Hokkaido UC population: 58.6 (19.0) years vs. 53.8 (14.2) years, respectively. For CD, male and female patients had a similar mean age in the Hokkaido population: 52.3 (15.2) years and 52.8 (16.4), respectively, whereas males were older than females in the Tokyo CD population; 61.4 (8.3) years vs. 52.2 (16.9) years, respectively. Male patients in their 60s represented the most predominant demographic in the Tokyo IBD population.

### 3.2. Calibration of Japanese Population of Patients with IBD

**Figure 4:**
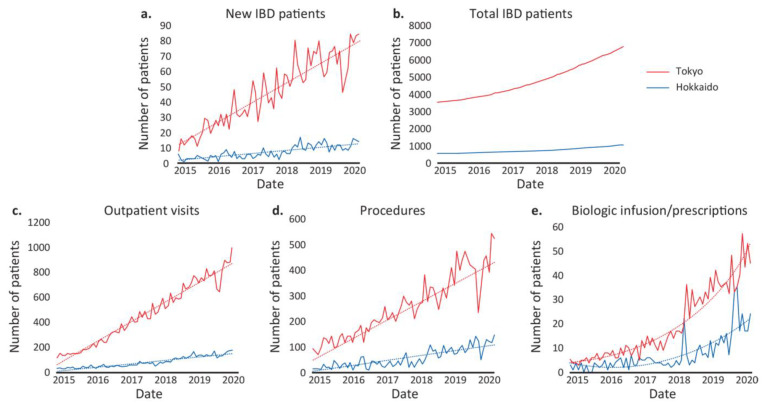
Calibration data with trend lines for patients with IBD during the pre-COVID-19 period in the Tokyo and Hokkaido populations for (**a**) new patients, (**b**) total patients, (**c**) outpatient visits, (**d**) procedures, and (**e**) biologics. IBD = inflammatory bowel disease.

There was an exponential growth in the Japanese IBD population during the pre-COVID-19 period (between 2015 and 2021) and an associated increase in demand and supply of healthcare services (Figure 4). There were increases in all the parameters (new patients, total patients, outpatient visits, procedures, and biologics) and these were more pronounced for the Tokyo population than for the Hokkaido population during this period. These increases in both regions were mainly driven by the diagnosis of new patients (Figure 4).

### 3.3. Impact of the COVID-19 Pandemic on Utilization of Medical Care for IBD

The first wave of the COVID-19 pandemic in April 2020 had the greatest impact on patient behaviors and medical care in IBD. Subsequent waves of the pandemic, although larger, did not have an equivalent impact.

**Figure 5:**
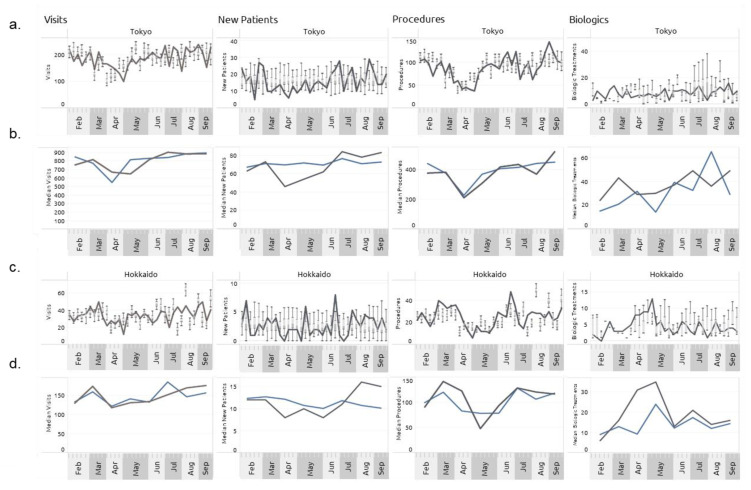
Impact of the COVID-19 pandemic in 2020 on key patient utilization of medical care for inflammatory bowel disease in Tokyo and Hokkaido. (**a**) Tokyo. Black line indicates weekly historical data. Box and whiskers indicate minimum, Q1, median, Q3, and maximum of weekly ensembles (50 runs) of model output. (**b**) Tokyo. Black line indicates monthly average historical data. Blue line indicates monthly average model output. (**c**) Hokkaido. Black line indicates weekly historical data. Box and whiskers indicate minimum, Q1, median, Q3, and maximum of weekly ensembles (50 runs) of model output. (**d**) Hokkaido. Black line indicates monthly average historical data. Blue line indicates monthly average model output.

Key points of disruption in patient behavior (demand) and medical care (supply) in the IBD population during the COVID-19 pandemic period included clinic visits for new patients seeking care, clinic visits for diagnosed patients receiving continuous care, time to diagnosis of new patients, number of procedures, interval between procedures, and interval between biologic prescriptions. Plotted in Figure 5 are weekly data of the patient behavior and medical care utilization for both Tokyo (Figure 5a,b) and Hokkaido (Figure 5c,d) IBD populations during the first (from 28 February 2020) and second (from 12 June 2020) waves of new COVID-19 cases (black lines), along with an ensemble (50 runs) of calibrated model runs (box and whisker plots). From Figure 5a,c it is observed that the disruption during the pandemic in Hokkaido was longer than in Tokyo. It was also noted that recovery in Hokkaido started later and was at a slower rate than recovery in Tokyo. The reduction in clinic visits in both areas corresponded with reductions in diagnosis of new patients and procedures. Biologic prescriptions decreased in Tokyo during the initial months of the pandemic but increased in Hokkaido during the same period. Plotted in Figure 5b,d are the monthly averages of the same data shown in Figure 5a,c. These results show that the averaged model output was able to capture the dynamics of patient visits and procedures well, but had a smaller response to the COVID-19 disruption for new patients and biologics compared with the actual data.

### 3.4. Interventions to Address the Impact of Disruptions

The interventions were designed to address the specific disruption and how they could alleviate these disruptions (supply and demand) for IBD medical services (Table 1). It was found that interventions (such as simple navigation assistance, barrier elimination, and emergency response infrastructure) that were designed to address supply side issues should be timed to address the immediate fall out from a disruption. This may mean that preventative measures or disaster recovery protocols need to be in place in preparation for such disruptions.

## 4. Discussion

In this PoC model, which used a computational simulation method, we found that both patient behavior (demand) and medical care (supply) were profoundly disrupted during the COVID-19 pandemic. We modeled the structural dynamics of the IBD treatment pathway and found the aspects of medical care that were most affected during the COVID-19 pandemic were time to diagnosis of new patients, clinic visits for new patients seeking care and diagnosed patients receiving ongoing care, number of procedures, and the interval between procedures or biologic prescriptions.

This model utilized a framework based on behavioral archetypes developed in the US but was matched to represent Japanese IBD populations in Tokyo and Hokkaido. This enabled a comparison of the impact of disruptions in an urban versus a regional setting in Japan. Calibration showed a reduction in the number of biologic prescriptions during the initial months of the COVID-19 pandemic in Tokyo, whereas prescriptions increased during the same period in Hokkaido followed by a sudden decrease, which likely indicated a sudden drop in supply. This disparity may reflect differences in stock stores and supply chains between urban and regional medical institutions in Japan. The calibration also showed that Hokkaido was slower to recover from disruptions to IBD care during the COVID-19 pandemic, indicating that institutions in Tokyo were better prepared or had more readily available resources to manage a pandemic situation. This suggests that the healthcare system in Tokyo was more resilient to this form of disruption despite the population density in the urban setting. Reasons for such differences are beyond the scope of our model, but may be explored using feedback-guided analysis, as shown by Tiongco and colleagues in their modeling of interdependence and the influence of stakeholder relationships and networks on resilience [22].

With the appropriation of the relevant data to localize the synthetic population, the current model can be applied to assess the impact of COVID-19 on IBD patient care in other regions of Japan. For example, Okinawa is an isolated island in Japan vulnerable to many visitors from outside the area (i.e., 3.7 million visitors in 2020 reported by Japanese municipality of Okinawa Prefecture). This unique geographical location may demonstrate different behavior changes by patients during the COVID-19 pandemic. The modeling framework may also be adapted to assess the influence of COVID-19 vaccination on patient behavior. New cases of COVID-19 are expected to decrease if nationwide vaccination is effective, and this may mitigate restrictions on patients’ clinical activities [23]. Moreover, this modeling framework can be reconfigured to assess the impact of COVID-19 on the management of patients with other chronic conditions such as HIV, diabetes, etc., for whom regular clinic visits, prescription refills, and access to medical facilities are also integral to the management of their condition [24,25,26,27]. The implementation of telemedicine has played an important role in the management of chronic conditions during the pandemic, and it is important to consider the contribution of this approach to patient care, particularly for patients who are anxious and have difficulty visiting health care facilities regularly [3].

The analysis of historical pandemic data showed that the initial COVID-19 wave, although small in both Tokyo and Hokkaido, created the most disruption. It is likely the declaration of a state of emergency and the resulting psychological impact on patients and healthcare providers influenced the reduction of patient visits and the number of exams/procedures. Subsequently, much larger waves of new COVID-19 cases, which were not accompanied by declarations of a state of emergency, had smaller impacts on the number of visits and exams/procedures likely as a result of fewer restrictions on activities and increased resilience of patients and healthcare providers who had adapted to the situation after the initial impact. Therefore, efforts to improve resilience may be most valuable if targeted at preparation for initial impacts. We observed a separation of immediate versus sustained impacts in the supply and demand structure. Owing to the impact on the human psyche, demand impact from a disruption may be ongoing and long-term. Alternatively, impact on supply may be more acute and shorter-term interventions, such as barrier elimination and emergency response infrastructure, can be effective.

Capacity constraints within the healthcare system need to be considered when proposing interventions to address disruptions. Analyses of historical data in our model showed that rapid alleviation of backlog caused by large disruptions creates oscillations in demand and supply due to the natural periodicity of clinic visits and treatments. Gradual recovery of demand avoids supply issues generated by large groups of patients requiring services at the same time, which are then followed by periods of low demand.

Prioritizing control of disease activity in patients with IBD in Japan during the COVID-19 pandemic has been emphasized by the Japan IBD COVID-19 Taskforce [10,28]. They have highlighted the importance of continuing current treatment to maintain remission or selecting an appropriate treatment to achieve prompt remission and emphasized that there is no need to discontinue immunomodulatory or biologic therapy. Moreover, consensus recommendations from The International Organization for the Study of Inflammatory Bowel Disease (IOIBD) state that the COVID-19 vaccine can be administered to patients with IBD while on maintenance biologic therapy or during induction with biologic therapy irrespective of timing within the treatment cycle [7]. Although not yet assessed, interventions, such as physician outreach and simple navigation assistance, may improve the impact on biologic prescriptions and help patients with IBD maintain stable disease. States of emergency have already been declared four times in Japan due to the COVID-19 pandemic, and each time, people’s activities and mobility have been greatly restricted. The super-aging society in Japan is well recognized, and there are concerns that ongoing restrictions will result in increased frailty rates and worsened frailty in the elderly [29]. It is therefore important to predict and discuss in advance how behavior will change in patients with IBD during future disruptions. Using historical data, the model will be configured to examine the magnitude of future surges in COVID-19 infections on IBD care in Japan.

The model will be further enhanced to simulate the impact of two disruptive scenarios on IBD medical care: a major earthquake depicting a spike interruption and a financial crisis depicting prolonged disruption. In the absence of historical data, we will enter hypothetical information. It is anticipated in these scenarios that there will be an initial spike in disruption of patient behaviors (demand) and medical care (supply) but with rapid recovery and a prolonged impact on patient behavior and medical care. These simulation analyses will be able to guide patients and healthcare providers to prepare for disruption from possible future crises and minimize the impact. The detailed results of this analysis will be reported elsewhere.

The outputs of our simulation model are limited by the data used. Owing to the small data samples, our results did not include metrics related to surgeries, and pre-biologic treatments could not be differentiated into separate categories (e.g., corticosteroids). The synthetic IBD population was created using data obtained before and during the COVID-19 pandemic, and the demand simulation was based on claims data that could only be collected up to 3 months before the start of data collection. In light of our rapidly evolving understanding of COVID-19 and the ever-changing epidemiology of the disease, outcome data may not reflect the current clinical situation. The model was not designed to provide insights about COVID-19 testing, vaccination, or infection in patients with IBD.

The purpose of this study was to evaluate the point-in-time exploration of how this methodology can be used at the current moment, during a health crisis. Moreover, the simulation model does not forecast future COVID-19 prevalence but uses likely specified scenarios as input; it needs to be regularly updated with current claims data and emerging clinical evidence to provide useful predictions and avoid errors in forecasting. Because the aim of the study was a proposal of methodology rather than a formal forecast, it can take several months to collect additional current data on patient healthcare utilization and conduct a formal validation. Hence, the focus of this manuscript is a general proposal of methodology with IBD as a demonstrative use case, and addressing the formal validation of this specific implementation of the methodology as a pragmatic forecast model will be covered as a separate topic in future.

This simulation model can be used by healthcare professionals to assess medical strategies in preparing better healthcare services for patients with IBD during disruptions, such as the COVID-19 pandemic. In addition, this simulation model can be applied in other disease conditions in which disruptions to healthcare services can impact patient prognosis.

The strengths of the present study include the agent-based modeling approach, which enabled each patient agent to have their own behavior related to seeking health services during the COVID-19 pandemic and to reactions to interventions. As the agents were distinct, there were no limitations on the distribution of agent characteristics (e.g., demographics or health history) that could be represented. In addition, the discrete-event modeling of the patient care pathway contained many complex features of the real patient experience that were supported by calibration to real-world data and can be updated and maintained in a way that is consistent with its structure.

The limitations of the present study include the scarcity of data at the start of the COVID-19 pandemic and shortly thereafter. The data used for the synthetic population of patient agents, while fitted to Japanese populations in Tokyo and Hokkaido, were based on data from US patients. Owing to small data sample sizes in the claims dataset, the results did not include metrics related to surgeries, and pre-biologic treatments were not divided into separate categories (e.g., steroids). Additionally, in the construction of the model, information and details were not included regarding the effects of COVID-19 testing, vaccination, or infection of patients with IBD.

In future studies, the present model could be further refined by using data from Japanese patients for the synthetic patient population, using updated claims data covering the most recent COVID-19 surge, examining seasonality in the healthcare system, and investigating capacity-side constraints to model supply changes. In addition, for the non-COVID-19 disruptions, the use of historical data could be used, in calibration, to generate a more realistic model response.

## 5. Conclusions

The Behavior Predictor and Demand Simulator can represent the dynamics of clinical care demand among patients with IBD in Japan, both in recapitulating historical demand curves and simulating future demand during disruption scenarios, such as pandemics, earthquakes, and economic crises.

## Figures and Tables

**Table 1:** Interventions to address the impact of disruptions on healthcare services and identify leverage points in the system.

Intervention	Description	Impact of Intervention on Supply		Impact of Intervention on Demand	
Physician outreach	Physicians message at a practice level to their patients (reach out to individual patients at risk)	N/A		15%	Patients starting to seek care; biologic prescriptions; clinic visits
System social signaling	Hospital systems reinforce confidence to their local area (targeted social media and local press efforts to a small footprint audience)	N/A		40%	Patients starting to seek care; biologic prescriptions; clinic visits
National confidence building	Government messages at a societal level, building confidence to stay engaged with care systems (broad scale media and high-level official communication efforts)	N/A		60%	Patients starting to seek care; biologic prescriptions; clinic visits
Simple navigation assistance	Education-focused initiatives on how best to navigate post-disruption (scheduling, where to go, who to see)	5%	Access to continuous care; access to biologics; supply of procedures	15%	Patients starting to seek care; biologic prescriptions; clinic visits
Barrier elimination	Partnership with government and/or private sector to subsidize transportation when regular channels are not available	40%	Access to continuous care; access to biologics; supply of procedures	40%	Patients starting to seek care; biologic prescriptions; clinic visits
Emergency response infrastructure	Infrastructure to provide emergency care on demand if the normal setting is disrupted	60%	Access to continuous care; access to biologics; supply of procedures	55%	Patients starting to seek care; biologic prescriptions; clinic visits

N/A = not applicable.

## Data Availability

The data underlying development of the platform are available in the article, references, and in the online supplementary material. Takeda does not plan to share data reported in this article based on the contract with sponsor PwC.

## Supplementary Materials

The following supporting information can be downloaded at: https://www.mdpi.com/article/10.3390/jcm12030757/s1, Figure S1: Schematic representation of the hierarchical model integration of the Behavior Predictor synthetic population, agent-based patient discrete event model, and population level system dynamics model. Figure S2: Schematic representation of patient-level discrete event submodels and their interdependencies. Table S1: Synthetic population fit for Tokyo and Hokkaido, data fields, population average values, and fitting errors. Table S2: IBD Severity Index, including factors and weights.
