# Supplementary material for: Computational Simulation Model to Predict Behavior Changes in Inflammatory Bowel Disease Patients during the COVID-19 Pandemic: Analysis of Two Regional Japanese Populations
Source: J Clin Med. 2023 Jan 18;12(3):757. doi: 10.3390/jcm12030757 (PMC9917920; doi:10.3390/jcm12030757)
Supplement: Supplementary file 1 [file jcm-12-00757-s001.zip › jcm-2105598-SI.pdf]

SUPPLEMENTAL MODEL METHODOLOGY

S1.) Behavior Predictor Synthetic Population

S1A.) Synthetic Population Localization to Japanese Data

The Behavior Predictor synthetic population contains 90,000 synthetic agents created by aggregating US data on care utilization, health conditions, behaviors, information and content preferences, product choices and usage, and motivators and stressors. These data were collected from a combination of consumer, geospatial, and behavioral surveillance data via statistical matching algorithms. Using linear programming, sample weights were calculated for each synthetic agent so that in aggregate the statistics of the synthetic population match the population data from the Japanese Ministry of Health, Labor and Welfare (MHLW) Health Statistics Office for 2016. Separate sample weight calculations were performed for Tokyo and Hokkaido. As shown in Table S1, errors were on the order of 10E-8 to 10E-10, indicating a close fit to the population data.

Table S1: Synthetic population fit for Tokyo and Hokkaido, data fields, population average values, and fitting errors

Constraint_Name	Tokyo Population Data (percent adult population)	Tokyo Synthetic Population Fit Errors (percent)	Hokkaido Population Data (percent adult population)	Hokkaido Synthetic Population Fit Errors (percent)
MALE BMI OVERWEIGHT	0.13328659	7.17E-09	0.19333879	7.66E-09
FEMALE BMI OVERWEIGHT	0.10388946	1.35E-08	0.13664278	2.36E-08
MALE BMI UNDERWEIGHT	0.02050563	2.95E-09	0.01708803	8.27E-09
FEMALE BMI UNDERWEIGHT	0.03224156	2.61E-09	0.04401228	1.09E-08
MALE BMI NORMAL WEIGHT	0.33443705	3.67E-09	0.27780246	1.03E-08
FEMALE BMI NORMAL WEIGHT	0.37563971	2.61E-09	0.33111566	6.73E-09
MALE NORMAL BLOOD PRESSURE	0.2472047	3.85E-10	0.19032667	4.29E-09
FEMALE NORMAL BLOOD PRESSURE	0.18346498	6.26E-09	0.14517743	2.46E-08
MALE SMOKER EVERYDAY	0.1339792	3.36E-09	NA	NA
FEMALE SMOKER EVERYDAY	0.04127183	3.23E-09	NA	NA
MALE SMOKER SOMETIMES	0.00908334	2.27E-09	NA	NA
FEMALE SMOKER SOMETIMES	0.00825437	6.57E-10	NA	NA
MALE SMOKER FORMER	0.06358335	8.57E-09	0.20074171	3.39E-09
FEMALE SMOKER FORMER	0.02063592	4.93E-08	0.07803315	2.40E-08
MALE NON SMOKER	0.2815834	1.59E-09	0.12353336	6.51E-09
FEMALE NON SMOKER	0.44160861	1.86E-09	0.37392536	7.20E-09

MALE NO ALCOHOLIC DRINKING	0.18308598	1.57E-09	0.17335677	3.91E-09
FEMALE NO ALCOHOLIC DRINKING	0.32811107	8.37E-10	0.34941588	3.62E-10
MALE NO REGULAR DENTIST VISIT	0.27931256	7.83E-10	0.07128147	3.15E-09
FEMALE NO REGULAR DENTIST VISIT	0.1981048	2.56E-09	0.04759468	6.32E-10
MALE DIAGNOSED DIABETES	0.07909314	1.12E-09	0.2332	7.41E-09
FEMALE DIAGNOSED DIABETES	0.09723644	2.43E-09	0.2653	5.20E-09
MALE NO DAILY EXERCISE	0.2332	2.29E-09	0.2557	1.45E-09
FEMALE NO DAILY EXERCISE	0.2653	1.17E-09	0.2326	2.15E-09
FAMILY SIZE 1	0.13	1.33E-09	0.13	2.70E-09
FAMILY SIZE 2	0.87	1.98E-10	0.87	4.03E-10
EMPLOYED	0.421855	5.40E-10	0.421855	2.04E-10
UNEMPLOYED	0.173145	5.44E-09	0.173145	1.36E-08
Age 21-29	0.13333333	2.44E-09	0.13333333	7.57E-09
Age 30-39	0.1769697	1.34E-09	0.1769697	4.63E-09
Age 40-49	0.16363636	9.15E-10	0.16363636	2.64E-09
Age 50-59	0.15878788	1.39E-09	0.15878788	2.29E-09
Age 60-69	0.1769697	2.44E-09	0.1769697	5.41E-09
Age 70-79	0.12242424	1.00E-09	0.12242424	1.44E-09
Age 80+	0.06787879	3.18E-09	0.06787879	1.07E-08

S1B.) Synthetic Population IBD Severity Index

In addition to the explicit data present in the Behavior Predictor synthetic population data on diagnosis of ulcerative colitis (UC) and Crohn disease (CD), an IBD Severity Index was created and evaluated for each patient agent. The necessity for this additional index was to identify agents in the patient pool who had not been diagnosed with IBD to date but who were at risk of becoming symptomatic during the future course of the simulation.

The IBD Severity Index was based on existing published indices for IBD [1,2] but modified to use the factors present in the Behavior Predictor data (see Table S2). For this reason, the index is not a rigorous measure of IBD risk but a qualitative way of identifying those patients most at risk for becoming symptomatic.

IBD Severity Index = sum(Severity Factor weights if present in a patient)

Table S2: IBD Severity Index, including factors and weights

Severity Factor	Weight
Bowel / colon cancer	0.05305039788
Stress	0.05305039788
Trouble falling asleep	0.05305039788
No or little exercise	0.05305039788
Smoking	0.6551724138
Low fat	0.05305039788
High protein	0.05305039788
Gastroenterologist visit	0.02652519894

S1C.) Inclusion of Synthetic Japanese IBD Patient Agents in Simulation Model

When the simulation model was initialized, the full synthetic population of patient agents were read in as data, including their patient ID, age, sex, severity, UC/CD status, and sample weights. At the start of a simulation, the patient agent pool was initialized by introducing randomly selected patients from the general synthetic population, including their sample weights. This was repeated until the sum of the patient pool sample weights equaled or exceeded the required patient pool population size at the start of the model. During the simulation model run, when the patient growth module indicated that new symptomatic patients were seeking care, new patients were introduced into the patient agent pool, and a similar process was followed to select new patients.

In this model, each patient agent represented a different number of real patients determined by their sample weight, as solved for in the fit to the MHLW Japanese population data (described in Section S1A).

S2.) Demand Simulator Agent-Based Patient Simulation Model

S2A.) Model Description

The model is an agent-based simulation model with a hierarchical structure (see Figure S1.). At the highest level it is a system dynamics model composed of a series of first order, possibly nonlinear, differential equations that numerically simulate the size of the IBD population forward in time using exogenously specified COVID-19 disruptions. The system dynamics model is numerical time stepped using an adaptive time stepped Runge-Kutta method.

Below this level is a discrete event model that is run in parallel to the system dynamics model. This discrete event model consists of a series of statecharts describing an agent's (also referred to here as a patient) trajectory through the treatment pathway. Many copies of this model are run simultaneously, one for each patient in the population. Information about the growth in population, which determines the introduction of new patients, and the current state of a disruptions' effect on healthcare supply and patient demand are communicated from the system dynamics model down to the patient discrete event models.

The model is presented in pseudocode as it is natively an algorithmic computational model. While the model can be expressed as formal mathematical expressions, the authors believe that presenting the description of the model in pseudocode is more efficient and makes the underlying processes of the model easier to comprehend.

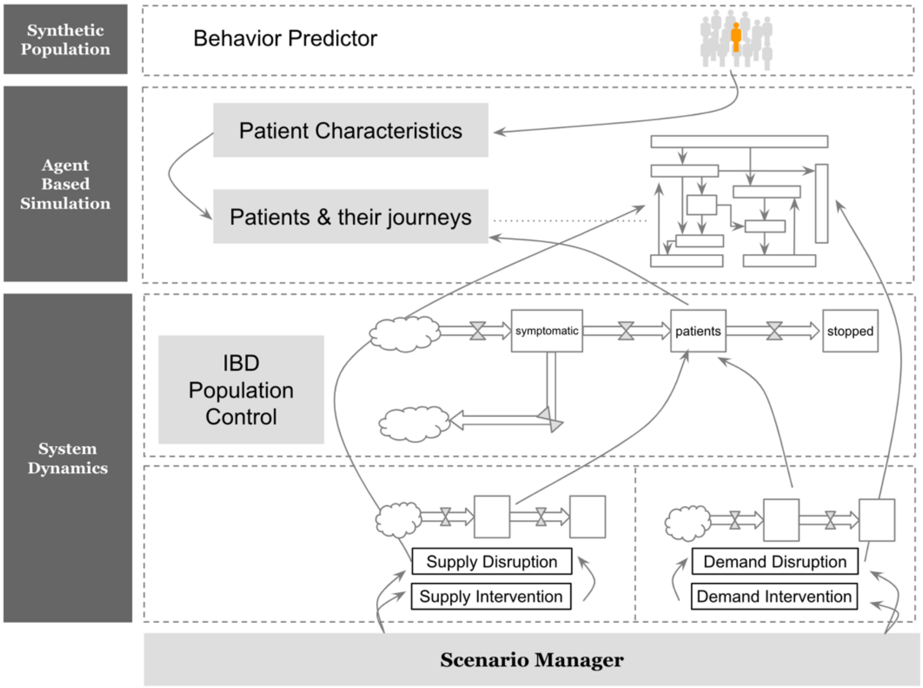

Figure S1: Schematic representation of the hierarchical model integration of the Behavior Predictor synthetic population, agent-based patient discrete event model, and population level system dynamics model.

Separate full versions of the model, including separate synthetic populations and calibrated parameters, were developed for Tokyo and Hokkaido. Both models were run for all scenarios and insights regarding the different responses to the COVID-19 pandemic in Tokyo and Hokkaido can be drawn from the differences in the model responses.

The simulation model explicitly represents the influence of disruptions, mainly the spread of the COVID-19 through Tokyo and Hokkaido, but it also supports other types of disruptions such as earthquakes and financial crises. While the response to the model has been rigorously calibrated to relevant COVID-19 pandemic data, the representation of other disruptions is largely idealized and qualitative. To represent the COVID-19 disruption more realistically, interventions to mitigate the intensity of the pandemic have also been included.
These include:

1. National confidence building
2. System social signaling
3. Physician outreach
4. Emergency response infrastructure
5. Barrier elimination
6. Simple navigation assistance

The details of these interventions are discussed in the main text in Section S2.4, Interventions to Address the Impact of Disruptions.

S2B.) Calibration

The full model was calibrated using a steepest descent (gradient descent) method with claims data from JMDC as described in the main text. There were two calibrations performed: one prior to the onset of COVID-19 in Japan running from 2008 to 2018, and a second during the first months of the COVID-19 pandemic in Japan that JMDC had data available for. The Tokyo and Hokkaido models were calibrated separately and have separate sets of parameters.

The following list of claims data from JMDC used to calibrate the model:

1. New patients
2. Visits
3. Procedures
4. Number of IBD treatments
5. Vedolizumab prescriptions for UC
6. Vedolizumab prescriptions for CD

A list of parameters that were calibrated to these data during either the historical (2008-2019) or COVID-19 era data can be found in Section S2E, Model Definitions, of this document.

S2C.) System Dynamics Model Equations and Pseudocode

IBD Population Dynamics

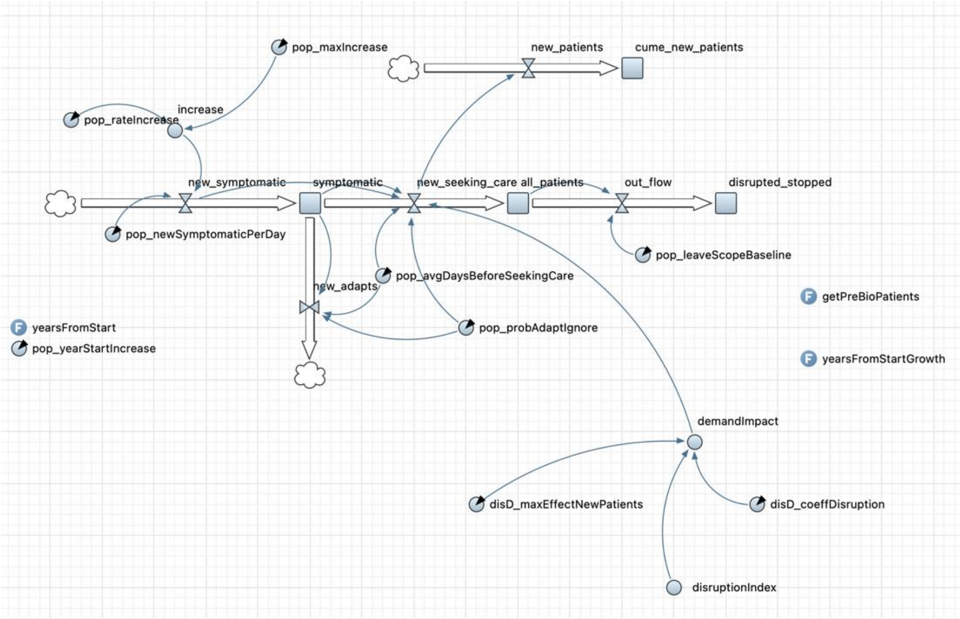


```
Δsymptomatic/Δt = + new_symptomatic - new_seeking_care - new_adapts

Δall_patients/Δt = + new_seeking_care - out_flow

Δdisrupted_stopped/Δt = + out_flow

Δcume_new_patients/Δt = new_patients

    new_symptomatic = pop_newSymptomaticPerDay * (1 +(pop_maxIncrease*10) * (1-
    1/exp(pop_rateIncrease*yearsFromStart())))

    new_adapts = (symptomatic * pop_probAdaptIgnore) / pop_avgDaysBeforeSeekingCare
```

```
new_seeking_care = delay3(new_symptomatic * demandImpact,
pop_avgDaysBeforeSeekingCare, (symptomatic / pop_avgDaysBeforeSeekingCare))
* (1.0-pop_probAdaptIgnore)

out_flow = all_patients * (pop_leaveScopeBaseline/10000)

new_patients = new_seeking_care
```

where delay3(input, delay time, initial value) is a third order exponential delay of the input.

Growth in Patients, Procedures, and Visits

In addition to the increase in the IBD population over time, the number of visits and services received by each patient also increased over time. To represent this, an additional growth term is included.

```
updateBioDueToGrowth()
    //if bio to start is greater than bio started, then create growth
    newBiosToday = new_bio_patients - bioStarted
    createdToday = 0.0

    //loop through until we have created enough people
    while createdToday < newBiosToday
            Pick a random Patient in pre-biologic treatment
            For that Patient, Generate message: "Start Bio"
            createdToday = createdToday + the Patient's population sample weight

    //update bioStarted to include the people we created today
    bioStarted = bioStarted + createdToday

yearsFromStartGrowth()
    //returns number years from 1975 that the model has been running


growthProcs = (growthProcs_maxIncrease) * (1-
1/exp(growthProcs_rateIncrease*yearsFromStartGrowth(growthProcs_year)))

growthVisits = (growthVisits_maxIncrease) * (1-
1/exp(growthVisits_rateIncrease*yearsFromStartGrowth(growthVisits_year)))

growthBio = (growthBio_maxIncrease/8000) * (1-
1/exp(growthBio_rateIncrease*yearsFromStartGrowth(growthBio_year)))

if growthBio > 0
then updateBioDueToGrowth()

Δnew_bio_patients /Δt = growthBio*m_treatment_pre_biologic

m_treatment_pre_biologic = number of patients in pre-biologic treatment multipled
                           by their sample weights
```

Disruption Dynamics

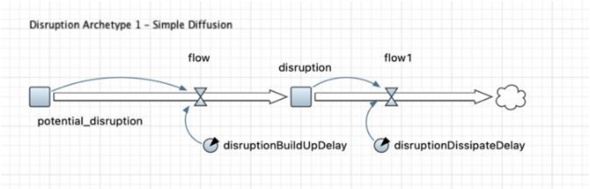


```
D(potential_dispution)/Δt = -potential_disruption / disruptionBuildUpDelay

Δ(disruption)/Δt = (potential_disruption / disruptionBuildUpDelay)
                    - (disruption / disruptionDissipateDelay)
```

Supply Dynamics

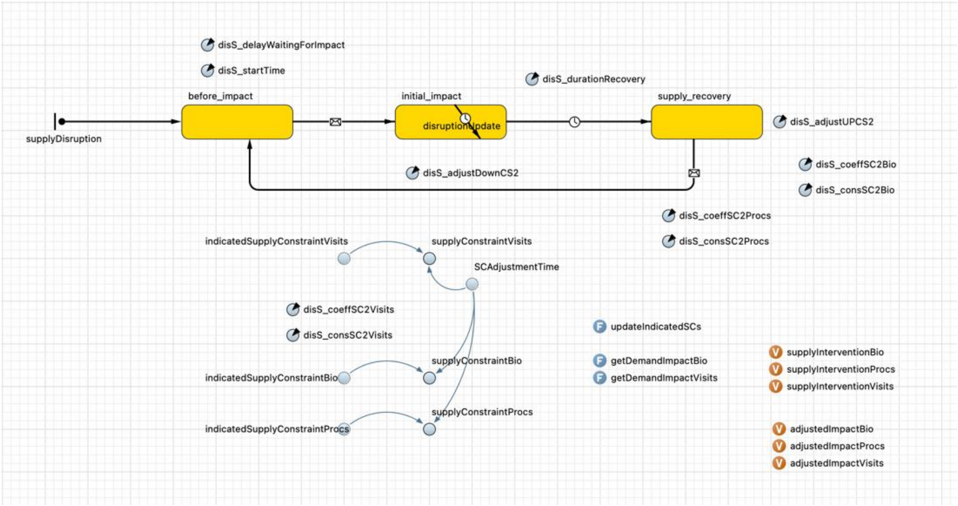

before_impact

```
indicatedSupplyConstraintVisits = 1
indicatedSupplyConstraintBio = 1
indicatedSupplyConstraintProcs = 1
SCAdjustmentTime = 1.0

if simTime == disS_startTime + disS_delayWaitingForImpact
then generate message "Start Supply Disruption"

Transition on message: "Start Supply Disruption"
```

initial_impact

```
//Time to reach max impact
SCAdjustmentTime = disS_adjustDownCS2

//Update supply constraints

indicatedSupplyConstraintVisits  = 1 – (1 – exp(disS_consSC2Visits -
disS_coeffSC2Visits*disruption)
/(1+exp(disS_consSC2Visits - disS_coeffSC2Visits*disruption))
*supplyInterventionVisits


indicatedSupplyConstraintBio = 1- (1 -  exp(disS_consSC2Bio -
disS_coeffSC2Bio*disruption)
/(1+exp(disS_consSC2Bio - disS_coeffSC2Bio*disruption)))
```

```
    *supplyInterventionBio

    indicatedSupplyConstraintProcs =  1 - (1 - exp(disS_consSC2Procs -
    disS_coeffSC2Procs*disruption)
    /(1+exp(disS_consSC2Procs - disS_coeffSC2Procs*disruption)))
    *supplyInterventionProcs

    transitionTime = disS_durationRecovery

supply_recovery
    indicatedSupplyConstraintVisits = 1
    indicatedSupplyConstraintBio = 1
    indicatedSupplyConstraintProcs = 1
    SCAdjustmentTime = disS_adjustUPCS2
    supplyInterventionBio = 1.0
    supplyInterventionProcs = 1.0
    supplyInterventionVisits = 1.0

    if simTime == disS_startTime + disS_delayWaitingForImpact
       + disS_durationRecovery + disS_recoveryTime
    then generate message "Return to before impact"

    Transition on message:  "Return to before impact"

supplyConstraintVisits = smooth3(indicatedSupplyConstraintVisits,SCAdjustmentTime,1)
supplyConstraintBio = smooth3(indicatedSupplyConstraintBio,SCAdjustmentTime,1)
supplyConstraintProcs = smooth3(indicatedSupplyConstraintProcs,SCAdjustmentTime,1)
```

Demand Dynamics

```
demandImpact = 1.0 - disD_maxEffectNewPatients * (1 - 1 / exp(disD_coeffDisruption
             * disruption))

disD_maxEffectNewPatients = disD_maxEffectNewPatients * demandInterventionPatients
disD_maxDemandBio = disD_maxDemandBio * demandInterventionBio
disD_maxDemandVisits = disD_maxDemandVisits * demandInterventionVisits
```

S2D.) Patient Level Discrete Event Model

Below the population-level system dynamics model, there is a parallel patient-level discrete event model that runs for each patient agent in the patient pool. This discrete event model consists of five submodels (statecharts) that the patients progress through and run in parallel (see Figure S2). The five submodels are as follows:

1. State of disease condition
2. State of continuous care
3. State of treatment: high level
4. State of treatment: vedolizumab
5. State of treatment: competitor biologic

Each is described with a diagram of event flow and pseudocode including a set of common functions that are used across the submodels.

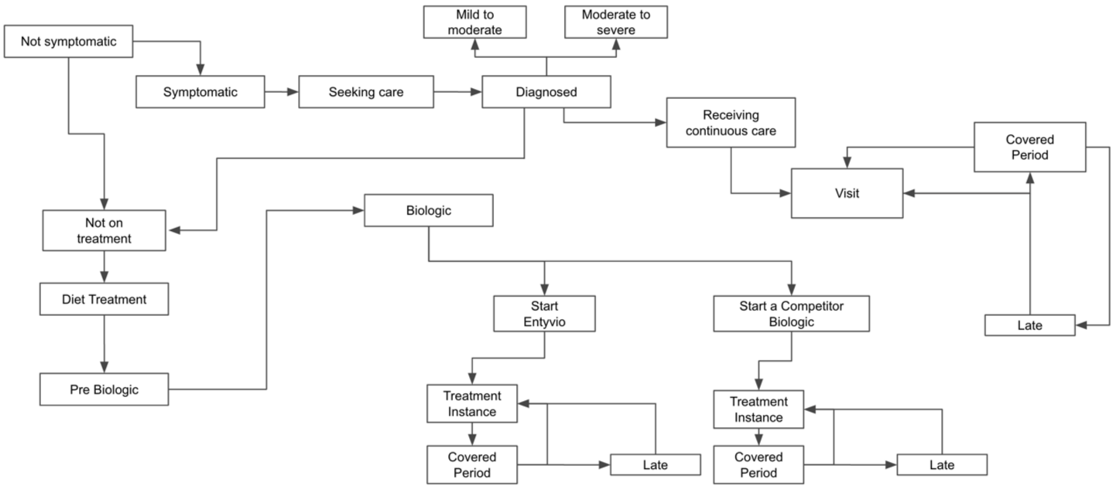

Figure S2: Schematic representation of patient-level discrete event submodels and their interdependencies

Patient-Level Discrete Event Model Common Functions

```
getDisruptionEffect(maxEffect,coeff) = 1.0 - maxEffect * (1 - 1 / exp(coeff
   * disruption))

updateSeverityStatus()
    severity = BPTwin.severity
    severityAdjusted = (severity - config_severityThreshold) * (1.0 / (1.0 -
       config_severityThreshold))
    probSevere = 0.0;

    if (severityAdjusted > p_severitySevereThreshold)
    then probSevere = (random() * 0.1) + 0.9

    severe = randomTrue(probSevere)

    if severe == true
    then isSevere = true

performProcedures(isDiagnosis)
    growth = 1.0

    if (!isDiagnosis)
    then growth = (1 + growthProcs)

    disruptionSupplyImpact = 1.0 - max(supplyConstraintProcs, 0.0);
    disruptionDemandImpact = 1.0 - max(getDisruptionEffect(disD_maxDemandVisits,
       disD_wDemandVisits), 0.0);
    disruptionImpact = 1.0 - (disruptionSupplyImpact + disruptionDemandImpact);

    //Update the values by procedure type

    if isDiagnosis == True //get procedures incurred during diagnosis
       scopesToday = p_procsPerDiagnosis * procedureShare_SCOPES* growth
    else  //if not diagnosis, then it's a normal visit
       scopesToday =  p_procsPerVisit * procedureShare_SCOPES* growth

    scopesToday = scopesToday *disruptionImpact
    scopesCume = scopesCume + scopesToday
```

```
if isDiagnosis == True //get procedures incurred during diagnosis
   bloodToday = p_procsPerDiagnosis * procedureShare_BLOOD* growth
else  //if not diagnosis, then it's a normal visit
   bloodToday =  p_procsPerVisit * procedureShare_BLOOD* growth

bloodToday =  bloodToday*disruptionImpact
bloodCume = bloodCume + bloodToday

if isDiagnosis == True //get procedures incurred during diagnosis
   stoolToday = p_procsPerDiagnosis * procedureShare_STOOL* growth
else  //if not diagnosis, then it's a normal visit
   stoolToday =  p_procsPerVisit * procedureShare_STOOL* growth

stoolToday = stoolToday* disruptionImpact
stoolCume = sStoolCume + toolToday

if isDiagnosis == True //get procedures incurred during diagnosis
   imageToday = p_procsPerDiagnosis * procedureShare_IMAGE* growth
else  //if not diagnosis, then it's a normal visit
   imageToday =  p_procsPerVisit * procedureShare_IMAGE* growth

imagingToday = imagingToday*disruptionImpact
imagingCume = imagingCume + imagingToday
```

State of Disease Condition

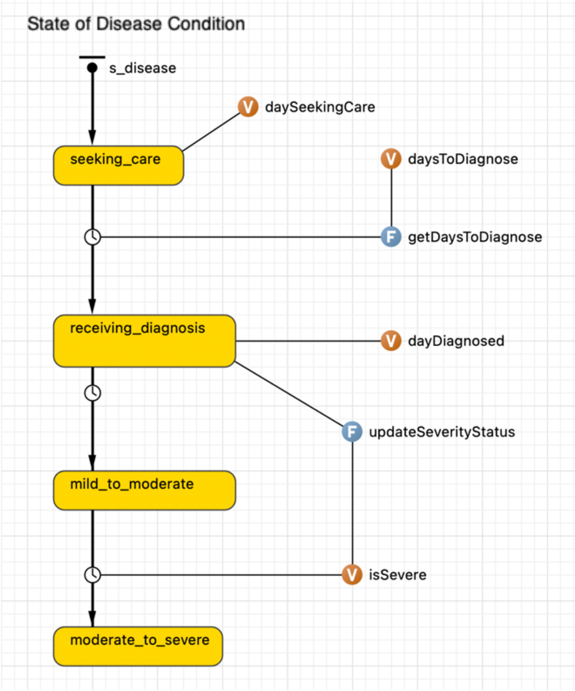


```
seeking_care
    daySeekingCare = simTime
    demandDaysDelayDiag = (daysToDiagnose/getDisruptionEffect(disD_maxDemandVisits,
         disD_wDemandVisits)) - daysToDiagnose
    supplyDaysDelayDiag = (daysToDiagnose/supplyConstraintVisits) - daysToDiagnose
    daysDelayDiag = max(demandDaysDelayDiag) + max(supplyDaysDelayDiag)

    transitionTime = daysDelayDiag

receiving_diagnosis
    dayDiagnosed = simTime
```

```
    //This person starts treatment and continuous care
    Generate message: "Requires CC"
    Generate message: "Start Diet Treatment"

    //Update severity
    updateSeverityStatus()

    //Procedures for diagnosis
    performProcedures(true)

    transitionTime = 0
```

mild_to_moderate

```
    if isSevere == True,
    then transitionTime = 0
```

moderate_to_severe

State of Continuous Care

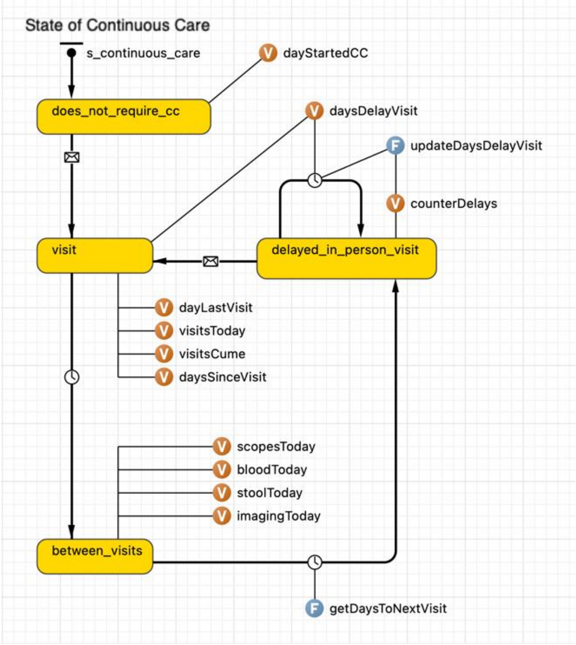

does_not _require_cc

```
    dayStartedCC = simTime

    transition on message: "Requires CC"
```

visit

```
    if dayLastVisit > 0
    then daysSinceVisit = simTime – dayLastVisit

    //Increment counters
    visitsToday++
    visitsCume++
    dayLastVisit = simTime

    //Update number of procedures performed
    //Procedures for diagnosis
    performProcedures(false)
```

```
    //Reset days delay visit
    daysDelayVisit = 0

    transitionTime = 1
```

between_visits

```
    scopesToday = 0
    bloodToday = 0
    stoolToday = 0
    imagingToday = 0

    thisDays = daysBetweenCCVisits * (1 - growthVisits)

    if in the State: mild_to_moderate
    then thisDays = thisDay*p_multVisitsIfMild

    transitionTime = random number sampled from triangular distribution with
    statistics:
       min = thisDays * mcLower
       max = thisDays * mcUpper
       mode = thisDays
```

delayed_in_person_visit

```
    //Check if patient will be late
    demandDaysDelayVisit =
              (daysBetweenCCVisits/getDisruptionEffect(disD_maxDemandVisits,
               disD_wDemandVisits)) - daysBetweenCCVisits
    supplyDaysDelayVisit = (daysBetweenCCVisits/supplyConstraintVisits) -
    daysBetweenCCVisits
    daysDelayVisit = min(90.0, demandDaysDelayVisit + supplyDaysDelayVisit)

    if daysDelayVisit < 1.0 || counterDelays > 3
    then generate message: "Go to visit"

    transition on message: "Go to visit"
```

State of Treatment: High Level

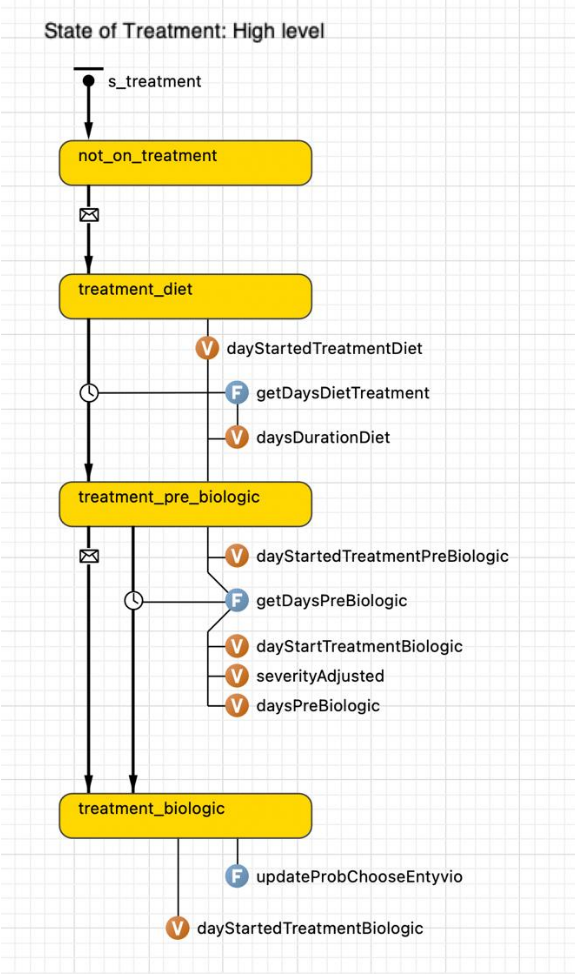


```
not_on_treatment
    transition on message: "Start Diet Treatment"

treatment_diet
    dayStartedTreatmentDiet = simTime
    transitionTime = daysDurationDiet

treatment_pre_biologic
    dayStartedTreatmentPreBiologic = simTime

    //If the date is before biologics start, then don't start this person

    if (severityAdjusted > p_biologicSeverityThresh)
    then
       //daysPreBiologic and daysDurationDiet are assumptions from the data
       //the day to start bio from starting treatment is
       //set to daysPreBiologic + daysDurationDiet

       numDaysPreBiologic = dayStartedTreatmentDiet + daysPreBiologic +
                            daysDurationDiet - dayStartedTreatmentPreBiologic;
       dayStartTreatmentBiologic = simTime + numDaysPreBiologic

    transitionTime = numDaysPreBiologic
    transition on message: "Start Bio"
```

```
treatment_biologic
    //Update time marker
    dayStartedTreatmentBiologic = simTime

    //Are we going to choose Vedo?
    //Uses the stock flow system on main to determine the Vedo share
    startVedo = randomTrue(probChooseVedo)

    if (startVedo)
    then generate message: "Start Vedo"
    else generate message: "Start Competitor Biologic"
```

State of Treatment: Vedolizumab

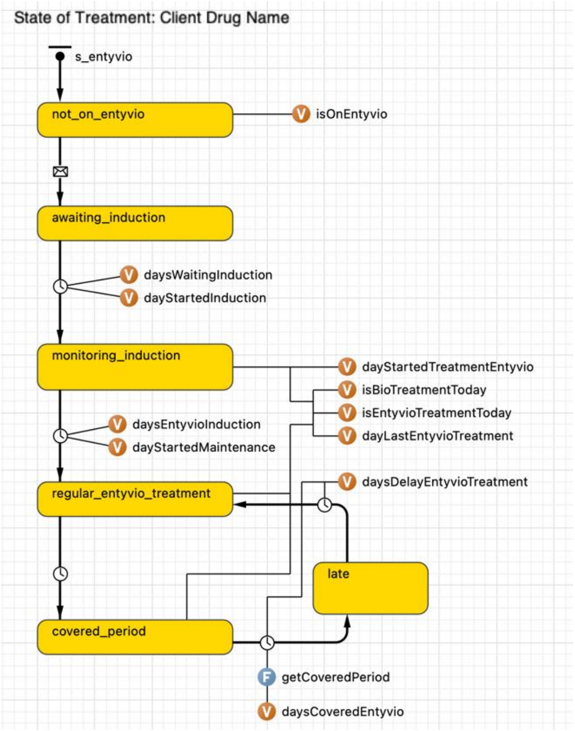


```
not_on_Vedo
    transition on message: "Start Vedo"

awaiting_induction
    isOnVedo = true
    transitionTime = daysWaitingInduction

monitoring_induction
    dayStartedInduction = simTime
    dayLastVedoTreatment = simTime
    isVedoTreatmentToday = true
    isBioTreatmentToday = true
    dayStartedTreatmentVedo = simTime

    transitionTime = daysVedoInduction

regular_Vedo_treatment
    dayStartedMaintenance = simTime
    dayLastVedoTreatment = simTime
    isVedoTreatmentToday = true
    isBioTreatmentToday = true
```

```
    transitionTime = 1
```

covered_period

```
    isVedoTreatmentToday = false
    isBioTreatmentToday = false

    transitionTime = daysCoveredVedo
```

late

```
    transitionTime = max(delayDueToDemand, delayDueToSupply) - 1.0
```

State of Treatment: Competitor Biologic

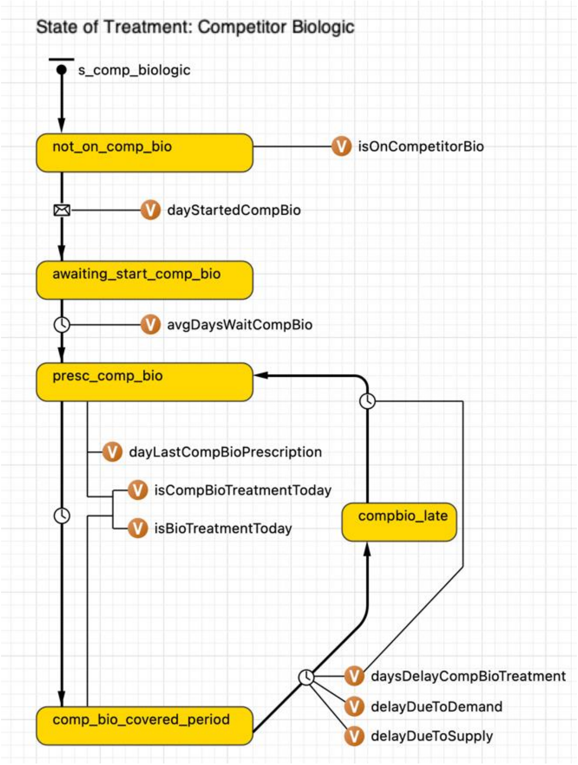

not_on_comp_bio

```
    transition on message: "Start Competitor Biologic"
```

awaiting_start_comp_bio

```
    isOnCompetitorBio = true
    dayStartedCompBio = simTime

    transitionTime = avgDaysWaitCompBio
```

presc_comp_bio

```
    //Update time of last prescription to today
    dayLastCompBioPrescription = simTime
    isCompBioTreatmentToday = true
    isBioTreatmentToday = true

    transitionTime = 1
```

comp_bio_covered_period

```
    //Record the treatment
    isCompBioTreatmentToday = false
```

```
    isBioTreatmentToday = false

    //Check if we're gonna be late
    demandEffect = getDisruptionEffect(disD_maxDemandBio, disD_wDemandBio)
    delayDueToDemand = (p_daysCompBioPrescription/demandEffect) -
    p_daysCompBioPrescription
    delayDueToSupply = (p_daysCompBioPrescription/supplyConstraintBio) -
    p_daysCompBioPrescription
    daysDelayCompBioTreatment = delayDueToDemand + delayDueToSupply
    transitionTime = p_daysCompBioPrescription

compbio_late
    transitionTime = daysDelayCompBioTreatment
```

S2E.) Simulation Model Definitions

Variables

bioStarted	variable	Growth
bloodCume	variable	Patient
bloodToday	variable	Patient.SoCC
counterDelays	variable	Patient.SoCC
createdToday	variable	Local
dayDiagnosed	variable	Patient.SoDC
dayLastCompBioPrescription	variable	Patient.SoTCB
dayLastVedoTreatment	variable	Patient.SoTV
dayLastVisit	variable	Patient.SoCC
daysCoveredVedo	variable	Patient.SoTV
daysDelayCompBioTreatment	variable	Patient.SoTCB
daysDelayDiag	variable	Local
daysDelayVisit	variable	Patient.SoCC
daySeekingCare	variable	Patient.SoDC
daysSInceVisit	variable	Patient.SoCC
dayStartedCC	variable	Patient.SoCC
dayStartedCompBio	variable	Patient.SoTCB
dayStartedInduction	variable	Patient.SoTV
dayStartedMaintenance	variable	Patient.SoTV
dayStartedTreatmentBiologic	variable	Patient.SoTHL
dayStartedTreatmentDiet	variable	Patient.SoTHL
dayStartedTreatmentPreBiologic	variable	Patient.SoTHL
dayStartedTreatmentVedo	variable	Patient.SoTV

daysVedoInduction	variable	Patient.SoTV
delayDueToDemand	variable	Patient.SoTCB
delayDueToSupply	variable	Patient.SoTCB
demandDaysDelayDiag	variable	Local
demandDaysDelayVisit	variable	Local
demandEffect	variable	Local
demandImpact	variable	Population
disruptionDemandImpact	variable	Local
disruptionImpact	variable	Local
disruptionSupplyImpact	variable	Local
growth	variable	Local
growthBio	variable	Growth
growthProcs	variable	Growth
growthVisits	variable	Growth
imagingCume	variable	Patient
imagingToday	variable	Patient.SoCC
indicatedSupplyConstraintBio	variable	Supply
indicatedSupplyConstraintProcs	variable	Supply
indicatedSupplyConstraintVisits	variable	Supply
isBioTreatmentToday	variable	Patient.SoTV
isCompBioTreatmentToday	variable	Patient.SoTCB
isOnCompetitorBio	variable	Patient.SoTCB
isOnVedo	variable	Patient.SoTV
isSevere	variable	Patient.SoDC
isVedoTreatmentToday	variable	Patient.SoTV
m_treatment_pre_biologic	variable	Main
newBiosToday	variable	Local
numDaysPreBiologic	variable	Local
probSevere	variable	Patient
SCAdjustmentTime	variable	Supply
scopesCume	variable	Patient
scopesToday	variable	Patient.SoCC
severityAdjusted	variable	Patient

simTime	variable	Main
startVedo	variable	Local
stoolCume	variable	Patient
stoolToday	variable	Patient.SoCC
supplyConstraintBio	variable	Supply
supplyConstraintProcs	variable	Supply
supplyConstraintVisits	variable	Supply
supplyDaysDelayDiag	variable	Local
supplyDaysDelayVisit	variable	Local
thisDays	variable	Local
visitsCume	variable	Patient.SoCC
visitsToday	variable	Patient.SoCC

Parameters

avgDaysWaitCompBio	parameter	Patient. SoTCB	Specified	Assumed	7	7
config_severityThreshold	parameter	Main	Calibrated	Historical	0.87	0.89
daysBetweenCCVisits	parameter	Patient	Specified	Assumed	30	30
daysDurationDiet	parameter	Patient. SoTHL	Specified	Assumed	367	461
daysPreBiologic	parameter	Patient. SoTHL	Specified	Assumed	501	299
daysToDiagnose	parameter	Patient. SoDC	Specified	Assumed	7	7
daysWaitingInduction	parameter	Patient. SoTV	Specified	Assumed	7	7
disD_coeffDisruption	parameter	Population	Calibrated	COVID	0.51	0.6
disD_maxDemandBio	parameter	Main	Calibrated	COVID	0.045	0.095
disD_maxDemandVisits	parameter	Supply	Calibrated	COVID	0.041	0.037
disD_maxEffectNewPatients	parameter	Population	Calibrated	COVID	0.11	0.27
disD_wDemandBio	parameter	Main	Calibrated	COVID	0.45	0.0015
disD_wDemandVisits	parameter	Supply	Calibrated	COVID	0.011	0.142
disruptionBuildUpDelay	parameter	Disruption	Specified	Scenario		
disruptionDissipateDelay	parameter	Disruption	Specified	Scenario		
disS_adjustDownCS2	parameter	Supply	Calibrated	COVID	79	26.656
disS_coeffSC2Bio	parameter	Supply	Calibrated	COVID	0.01	0.47

disS_coeffSC2Procs	parameter	Supply	Calibrated	COVID	0.004	0.078
disS_coeffSC2Visits	parameter	Supply	Calibrated	COVID	0.012	0.003
disS_consSC2Bio	parameter	Supply	Calibrated	COVID	4.642	41.1
disS_consSC2Procs	parameter	Supply	Calibrated	COVID	0.968	1.76
disS_consSC2Visits	parameter	Supply	Calibrated	COVID	1.52	3.14
disS_delayWaitingFor Impact	parameter	Supply	Calibrated	COVID	12.426	64.5
disS_durationRecovery	parameter	Supply	Calibrated	COVID	61.568	31.725
disS_recoveryTime	parameter	Supply	Specified	Scenario		
disS_startTime	parameter	Supply	Specified	Scenario		
growthBio_maxIncrease	parameter	Growth	Calibrated	Historical	0.33	0.7
growthBio_rateIncrease	parameter	Growth	Calibrated	Historical	0.386	0.73
growthBio_year	parameter	Growth	Calibrated	Historical	40	40.5
growthProcs_maxIncrease	parameter	Growth	Calibrated	Historical	0	0.49
growthProcs_rateIncrease	parameter	Growth	Calibrated	Historical	0.24	0.33
growthProcs_year	parameter	Growth	Calibrated	Historical	41	40.46
growthVisits_maxIncrease	parameter	Growth	Calibrated	Historical	0.51	0.39
growthVisits_rateIncrease	parameter	Growth	Calibrated	Historical	0.729	0.5
growthVisits_year	parameter	Growth	Calibrated	Historical	40.02	40.8
mcLower	parameter	Patient	Specified	Assumed	0.8	0.8
mcUpper	parameter	Patient	Specified	Assumed	1.2	1.2
p_biologicSeverityThresh	parameter	Main	Calibrated	Historical	0.938	0.755
p_daysCompBioPrescription	parameter	Main	Calibrated	Historical	67.503	70
p_multVisitsIfMild	parameter	Main	Calibrated	Historical	0.57	0.602
p_procsPerDiagnosis	parameter	Main	Calibrated	Historical	2.54	2.536
p_procsPerVisit	parameter	Main	Calibrated	Historical	0.57	0.602
p_severitySevereThreshold	parameter	Main	Calibrated	Historical	0.805	0.782
pop_avgDaysBeforeSeekingCare	parameter	Population	Calibrated	Historical	14	14
pop_leaveScopeBaseline	parameter	Population	Calibrated	Historical	0.001	0.001
pop_maxIncrease	parameter	Population	Calibrated	Historical	6.277	4.641
pop_newSymptomaticPerDay	parameter	Population	Calibrated	Historical	0.055	0.0083
pop_probAdaptIgnore	parameter	Population	Calibrated	Historical	0	0
pop_rateIncrease	parameter	Population	Calibrated	Historical	0.12	0.19657

probChooseVedo	parameter	Patient.<br>SoTHL	Specified	Assumed	0.277	0.277
procedureShare_BLOOD	parameter	Main	Calibrated	Historical	0.45	0.45
procedureShare_IMAGE	parameter	Main	Calibrated	Historical	0.09	0.09
procedureShare_SCOPE	parameter	Main	Calibrated	Historical	0.25	0.25
procedureShare_STOOL	parameter	Main	Calibrated	Historical	0.21	0.21
severity	parameter	Patient	Specified	Behavior<br>Predictor	per<br>Patie<br>nt	per<br>Patien<br>t

Stocks & Flows

Name	Type	Location	Initial Condition: Jan 1, 2020	Initial Conditions: Jan 1, 2020
			Tokyo	Hokkaido
all_patients	stock	Population	3554	647
cume_new_patients	stock	Population	0	0
disrupted_stopped	stock	Population	0	0
disruption	stock	Disruption	0	0
new_adapts	flow	Population	NA	NA
new_bio_patients	stock	Growth	0	0
new_patients	flow	Population	NA	NA
new_seeking_care	flow	Population	NA	NA
new_symptomatic	flow	Population	NA	NA
out_flow	flow	Population	NA	NA
potential_disruption	stock	Disruption	Scenarios: Total Magnitude	Scenarios: Total Magnitude
symptomatic	stock	Population	27	5

Function Arguments

coeff	argument	Local
isDiagnosis	argument	Local
isUC	argument	Local
maxEffect	argument	Local

Interventions

				Source	NATIONAL CONFIDENCE BUILDING	SYSTEM SOCIAL SIGNALING	PHYSICIAN OUTREACH	EMERGENCY RESPONSE INFRASTRUCTURE	BARRIER ELIMINATION	SIMPLE NAVIGATION ASSISTANCE
demandIntervention Patients	variable	Main	Specified	Scenario	0.4	0.6	0.85	0.45	0.4	0.85
demandInterventionBio	variable	Main	Specified	Scenario	0.4	0.6	0.85	0.45	0.4	0.85
demandIntervention Visits	variable	Main	Specified	Scenario	0.4	0.6	0.85	0.45	0.4	0.85
supplyIntervention Bio	variable	Supply	Specified	Scenario	1	1	1	0.4	0.4	0.95
supplyIntervention Procs	variable	Supply	Specified	Scenario	1	1	1	0.4	0.4	0.95
supplyIntervention Visits	variable	Supply	Specified	Scenario	1	1	1	0.4	0.4	0.95

Messages

"Go to visit"	message	Patient.SoCC
"Requires CC"	message	Patient.SoDC
"Return to before impact"	message	Supply
"Start Bio"	message	Growth
"Start Competitor Biologic"	message	Patient.SoTHL
"Start Diet Treatment"	message	Patient.SoDC
"Start Supply Disruption"	message	Supply
"Start Vedo"	message	Patient.SoTHL

S2F.) Simulation Model Input/Output Data and Scenario Settings

A list of data used as input during simulation runs can be found in Supplemental Table S1

A list of model output generated during simulation runs can be found in Supplemental Table S2

Supplemental References

1. Kumar, M.; Garand, M.; Al Khodor, S. Integrating omics for a better understanding of Inflammatory Bowel Disease: a step towards personalized medicine. *Journal of Translational Medicine*, **2019**, 17, 419.

2. Abegunde, A.T.; Muhammad, B.H.; Bhatti, O.; Ali T. Environmental risk factors for inflammatory bowel diseases: evidence based literature review. *World J Gastroenterol* **2016**, 22(27), 6296–6317.
